# Case report: Isolated eyelid metastasis of ccRCC 5 years after receiving radical nephrectomy

**DOI:** 10.3389/fonc.2024.1321919

**Published:** 2024-03-15

**Authors:** Huaqi Yin, Zheng Du, Jiuwen Zhang, Yongkang Ma, Shiming Zhao, Tiejun Yang

**Affiliations:** ^1^Department of Urology, The Affiliated Cancer Hospital of Zhengzhou University, Henan Cancer Hospital, Zhengzhou, China; ^2^Department of Urology, The Affiliated People’s Hospital of Zhengzhou University, Henan provincial Hospital, Zhengzhou, China; ^3^Shanghai Jiao Tong University School of Medicine, Shanghai, China

**Keywords:** renal cell carcinoma, eyelid metastasis, renal tumor, sunitinib, MRI

## Abstract

**Introduction:**

The most common sites of clear cell renal cell carcinoma(ccRCC) metastasis are the lung, bones, liver and brain; eyelid metastasis is a rare occurrence.

**Case presentation:**

We report a case of ccRCC metastasis to the left eyelid after radical nephrectomy, and remission after sunitinib treatment.

**Conclusions:**

Although the probability of eyelid metastasis rate is very low, tumor metastasis to the eyelid skin is possible after radical nephrectomy. Therefore, any rash like changes on the skin during the review procedure cannot be ignored by the physician.

## Introduction

Renal cell carcinoma (RCC) is the most common type of adult kidney cancer, and clear cell renal cell carcinoma (ccRCC) represents the most common renal cancer histology. About two-thirds of patients have no metastases when they are first diagnosed (1). Patients without metastatic ccRCC can be cured by nephrectomy. However, more than one third of patients with stage II-III ccRCC have a recurrence after radical nephrectomy (2), metastasis is common in lung, bone, liver, brain and so on (3). Metastatic sites of clear cell renal cell carcinoma are generally characterized by good blood supply and weak autoimmune function, such as lung and bone. The skin appears to be an exempt site for ccRCC metastasis. In this paper, we report a case of ccRCC metastasis to the left eyelid 5 years after radical nephrectomy and remission after targeted therapy with sunitinib.

## Case presentation

**Figure 1:**
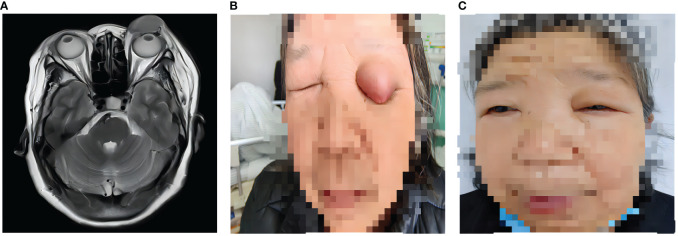
The change of ccRCC metastasis on the left eyelid. **(A)** MRI image of the mass on the left eyelid. **(B)** A painful mass on the left eyelid. **(C)** The mass was disappeared after 1 months of sunitinib treatment.

A 59-year-old woman was admitted to the hospital with the chief complaint of a 2-month left eyelid mass with pain. Physical examination revealed a mass on to the left eyelid. 5 years ago, the patient accidentally discovered a mass on the right side of his abdomen. She had no symptoms of hematuria, frequent urination, or painful urination, nor was she accompanied by dysuria or low back pain. The patient had undergone laparoscopic radical nephrectomy of ccRCC in September 2018, and the TNM stage was T2N0M0. Pathological diagnosis: clear cell renal cell carcinoma, invasion of the renal capsule, WHO/ISUP grade III, no cancer cells found in the ureteral resection margin. Considering the patient’s medical history, we thought that the eyelid mass might be the result of ccRCC metastasis. MRI examination suggested the possibility of metastatic tumor (**Figure 1A**). After ruling out the remaining possibilities, we recommended that the patient undergo eyelid mass resection to remove the metastatic tumor. However, the patient and her family refused operation and biopsy. The patient decided not to use cabozantinib due to financial difficulties, we jointly decided to use sunitinib for antineoplastic therapy. She was medically treated after signing an informed consent form. After 1 months of treatment, the mass was significantly decreased compared with before (**Figures 1B, C**), and the pain disappeared.

## Discussion

This case of eyelid metastasis 5 years after radical nephrectomy without accompanying metastasis in other locations proved that RCC can have unusual sites of metastasis. So far, there are few cases of ccRCC metastasis to the eyelid reported worldwide. In most cases, eyelid metastases usually occur later in the disease course as a manifestation of systemic metastatic cancer, and sometimes may be the first manifestation when the origin of the tumor is unknown (4). It has been reported that patients with RCC with isolated eyelid metastasis can obtain good prognosis after undergoing simultaneous resection of the primary and metastatic tumors. Nima Mikail et al. reported that simultaneous resection of the primary RCC and eyelid metastases did not result in tumor progression during a follow-up period of 2 years (5).

For metastatic ccRCC, targeted therapy alone or combined with immunotherapy has achieved efficacy and improved the overall survival time of patients (6). However, there is still a lack of consensus on the routine adjuvant therapy for patients with isolated eyelid metastasis after metastasis resection. In some reports, patients with eyelid metastasis were treated with resection, radiotherapy or interferon therapy, but some had poor prognosis (7, 8). According to some reports, the patient’s symptoms were relieved and the patient’s life quality was greatly improved after the use of sunitinib (9, 10).

Although eyelid metastasis from ccRCC is rare, cases of skin metastases have been reported. Our study reviewed the literature on skin metastases of renal cell carcinoma (**Table 1**). Therefore, postoperative patients with ccRCC should be regularly reexamined with chest CT, liver, gallbladder, pancreas, spleen and kidney color Doppler ultrasound. In addition, skin rashes or nodules on the surface of the skin, especially painless lesions should be dynamically monitored to early find metastasis. Some scholars have pointed out that RCC-Ma, a ccRCC marker, has specificity for ccRCC skin metastasis, which is also worthy of reference in our subsequent research and application (19).

**Table 1:** Literature reports about skin metastasis of cell renal carcinomas.

Article name	gender	age	metastasis site	radical nephrectomy of the kidney	treatment protocol
Metástasis palpebral como primera manifestación de un tumor renal (4)	male	87	eyelid	No	Surgery
An Atypical Cutaneous Metastasis in a Case of Clear Cell Renal Carcinoma (11)	male	52	scalp	Yes	Surgery
Eyelid Metastasis as the Initial Presentation of a Renal Cell Carcinoma (12)	male	77	eyelid	No	Surgery and pazopanib.
Cutaneous metastasis of renal cell carcinoma (9)	female	72	Facial skin, bilateral thighs, and lungs	Yes	Sunitinib
Eyelid metastasis as the initial presentation of renal cell carcinoma: Case report (13)	male	62	eyelid	No	Surgery
Severe bleeding eyelid after trivial trauma: conjunctival metastasis of a renal cell carcinoma (14)	female	70	eyelid	No	Surgery
Unilateral Blepharoptosis from Renal Cell Carcinoma (15)	male	47	eyelid	No	Unknown
Metastasis to the Eye and Orbit from Renal Cell Carcinoma—A Report of Three Cases and Review of Literature (7)	male	67	iris	Yes	Radiotherapy and alfa-interferon
	male	58	left eye diffuse infiltration	No	Surgery
	female	23	right orbital	Yes	Unknown
A case of an orbital metastasectomy in a renal cell carcinoma after sunitinib treatment: a case report (10)	male	81	orbital	Yes	Sunitinib
Eyelid-Sparing Adjuvant Radiation Therapy for Renal Cell Carcinoma (16)	male	63	eyelid	Yes	Surgery and external beam radiation therapy
Lacrimal Sac Metastases from Renal Cell Carcinoma (17)	female	56	lacrimal sac	Yes	Surgery
Ocular Metastatic Renal Carcinoma Presenting with Proptosis (18)	female	73	orbital	Yes	Surgery

## Conclusion

After radical nephrectomy for ccRCC, there is the possibility of metastasis to the eyelid. In the follow-up process after surgery, some lesions or nodules of the body epidermis should not be ignored.

## Data availability statement

The raw data supporting the conclusions of this article will be made available by the authors, without undue reservation.

## Ethics statement

Written informed consent was obtained from the individual(s) for the publication of any potentially identifiable images or data included in this article.

## Author contributions

YH: Conceptualization, Data curation, Formal Analysis, Investigation, Methodology, Writing – original draft, Project administration, Software, Writing – review & editing. DZ: Writing – review & editing, Conceptualization, Data curation, Formal analysis, Investigation, Writing – original draft. ZJ: Conceptualization, Data curation, Formal analysis, Methodology, Writing – original draft. MY: Conceptualization, Data curation, Formal analysis, Investigation, Methodology, Writing – original draft. ZS: Data curation, Formal analysis, Investigation, Methodology, Writing – original draft. YT: Project administration, Supervision, Writing – review & editing.
